# Serum Chloride Is Inversely Associated With 3 Months Outcomes in Chinese Patients With Heart Failure, a Retrospective Cohort Study

**DOI:** 10.3389/fcvm.2022.855053

**Published:** 2022-04-28

**Authors:** Zhiqing Fu, Li An, Xiaochun Lu, Li Sheng, Hongbin Liu

**Affiliations:** ^1^Department of Cardiology, The Second Medical Center & National Clinical Research Center for Geriatric Diseases, Chinese PLA General Hospital, Beijing, China; ^2^Department of Respiratory and Critical Care Medicine, The Second Medical Center & National Clinical Research Center for Geriatric Diseases, Chinese PLA General Hospital, Beijing, China

**Keywords:** serum chloride, prognosis, all-cause death, rehospitalization, heart failure

## Abstract

**Background:**

Serum chloride was recently found to be associated with prognosis of heart failure in western countries. However, the evidence was scarce in Asia. We aimed to investigated the relationship between serum chloride and clinical outcomes in a Chinese cohort with hospitalized heart failure.

**Methods:**

We retrospectively analyzed the data from PhysioNet, involving 1996 patients who were admitted with heart failure between December 2016 and June 2019. Outcome was a composite endpoint of all-cause death or rehospitalization at 3 months.

**Results:**

The incidence of the composite endpoint was 26.8% (535/1,996); it was 32.2% (213/662), 25.0% (165/661), and 23.3% (157/673) by chloride tertiles (from the lowest to the highest), respectively. The serum chloride at admission was independently and inversely associated with the composite endpoint risk (hazard ratio: 0.967; 95% confidence interval: 0.939 to 0.996; *p* = 0.026) in contrast to sodium, which was no longer significant (*p* > 0.05) after multivariable adjustment. Pearson correlation between serum chloride and sodium was 0.747 (*p* < 0.001). However, an increased AUC was not observed by adding sodium to model composed of age, sex, NYHA class, diabetes, log BNP and chloride (0.620 *vs*. 0.612, *p* = 0.132). Subgroup analysis showed the presence or absence of hyponatremia did not affect the association between chloride and composite endpoint risk.

**Conclusions:**

Low serum chloride at admission was associated with poor outcomes in Chinese hospitalized patients with heart failure. These findings warrant future studies for tackling the potential pathophysiological mechanisms and correction methods of hypochloremia in heart failure.

## Introduction

Heart failure is a leading cause of hospitalization in the elderly (1). Data suggests that the incidence of heart failure is mostly flat or declining but that the burden of mortality and hospitalization remains mostly unabated despite significant ongoing efforts to treat and manage heart failure (2). Nearly 80% of heart failure cost is due to hospitalization and readmission (3, 4). Early identification of patients at high-risk heart failure may allow for a more rapid optimization of therapy to symptoms' improvement and patient survival.

The main cause of heart failure readmission is exacerbated congestion due to neuroendocrine hyperactivation and fluid retention (5). Accumulating evidence suggests that serum sodium is an important determinant of fluid retention in heart failure (6, 7). Diuretics is the current cornerstone of decongestive treatment. However, available diuretics do not improve the long-term prognosis of heart failure (8, 9). Frequent diuresis can also lead to serious electrolyte disturbances. One of the electrolyte disorders that has been overlooked in the past is abnormal chloride homeostasis. In recent years, the pathophysiological role of serum chloride - an important anion in the body - has been gradually recognized in heart failure. It has been demonstrated that low serum chloride levels are associated with a poor prognosis in heart failure (10–12). However, most of the available evidence is focused on Europe and America, with only a few studies from Japan in Asia (13, 14) and little from China (15).

Therefore, we conducted a study in a Chinese population to examine the association of serum chloride with mortality and readmission rates.

## Materials and Methods

### Study Population

A retrospective single-center database (16), which was interrogated in the PhysioNet (17), was established regarding the characteristics of heart failure patients in China by integrating electronic healthcare records and follow-up outcome data. The data collected information for a total of 2,008 adult patients with heart failure at Zigong Fourth People's Hospital, Sichuan, China from December 2016 to June 2019, and was approved by the ethics committee of Zigong Fourth People's Hospital (Approval Number: 2020-010). Informed consent was waived due to the retrospective design of the study. The study complies with the Declaration of Helsinki. Only the first admission for a patient was included in the cohort if they were subsequently readmitted. Data on subsequent hospital admissions and mortality were obtained at mandatory follow-up visit at 3 months (if the patient was unable to reach the clinical center, the follow-up visit was replaced by a telephone call).

Heart failure was defined according to the European Society of Cardiology (ESC) criteria (18). Data collected for the dataset included six categories: demographic data, baseline clinical characteristics, comorbidities, laboratory findings, drugs and outcomes.

Hypochloremia, hyponatremia and hypokalemia was defined as greater than two standard deviations (SD) below the mean electrolyte level in the normal distribution for the local population (<99 mmol/L, <137 mmol/L, and <3.5 mmol/L, respectively).

### Outcomes

The outcome was the composite endpoint of all-cause death or rehospitalization at 3 months. Additional outcomes analyzed were all-cause death, and rehospitalization, respectively.

### Statistical Analysis

Categorical data are presented as percentages, normally distributed continuous data are presented as mean ± SD and non-normally distributed variables are presented as median and interquartile range (IQR). Log-base-2-transformed BNP was used. Glomerular filtration rates were estimated by incorporating creatinine into the Chronic Kidney Disease Epidemiology Collaboration (CKD-EPI) formula. The cohort was split into tertiles of serum chloride. For baseline characteristics analysis, the statistical differences among tertiles of serum chloride were tested with one-way ANOVA for continuous variables and chi-square test for categorical variables. The smooth curve fitting was used to identify non-linear relationships between serum chloride and outcome. Hazard ratios (HR) were estimated by Cox proportional-hazards models, with adjustment for potential confounding factors. Univariable analysis was conducted using all variables in the dataset and variables with *p* > 0.1 in univariable analysis or with more than 10% missing values (an arbitrary threshold) were not included in the multivariable analyses. Kaplan-Meier curves were used to demonstrate outcome by different groups. Cumulative mortality rates were compared by log-rank analysis. The model covariates were selected a priori (based on previous prognostic reports in patients with heart failure and clinical experience) either because of their prognostic relevance or their potential to confound the chloride-risk relationship. These included age, sex, systolic blood pressure, diabetes, New York Heart Association (NYHA) class I/II vs. III/IV, LVEF, as well as serum sodium, serum carbon dioxide combining power (CO_2_CP), blood urea nitrogen and eGFR (19). Receiver operating characteristic (ROC) curve analysis was used to estimate the predictive value of serum chloride and sodium for composite endpoint risk in heart failure patients. Pearson correlation coefficients were used to show correlations between serum chloride and sodium. The subgroup analyses were performed using stratified Cox proportional hazards models. Interaction among subgroups was inspected by the likelihood ratio test. All of the analyses were performed with the statistical software packages R 3.3.2 (http://www.R-project.org, The R Foundation) and Free Statistics software versions 1.3. Double-sided *p*-values <0.05 were considered statistically significant.

## Results

### Baseline Characteristics

**Figure 1:**
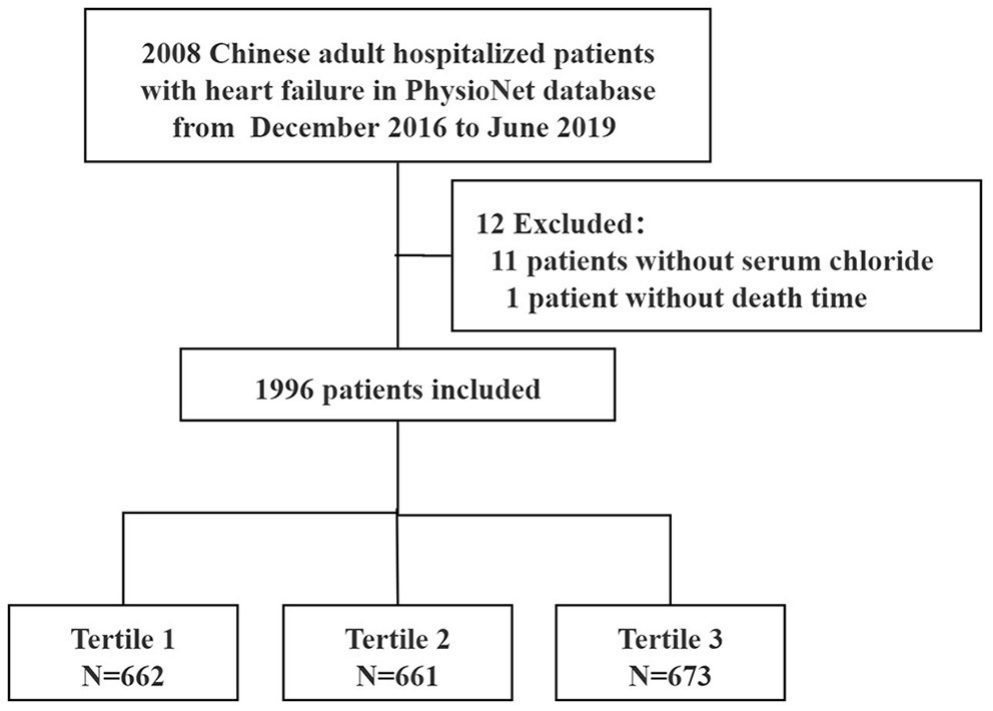
Flow chart of patients selection.

Of the initial 2008 patients, 12 were excluded because of missing values for serum chloride on admission (*n* = 11) or death time (*n* = 1). Thus, 1996 patients were finally included in the analysis (Figure 1). 91.1% participants were older than 60 years and 42.1% were males. The prevalence of hypochloremia was 26.1% (521/1996) in this cohort with heart failure.

The cohort was split into three groups across the serum chloride tertile. The baseline characteristics are shown in Table 1. Median chloride of patients in the first tertile (tertile 1) was 95.2 mmol/L (range 70.9–100 mmol/L) and 107.6 mmol/L (range 104.8–125.1 mmol/L) in the third tertile (tertile 3).

**Table 1:** Baseline characteristics according to chloride tertiles.

**Variables**		**Chloride (mmol/L)**			
	**Total** **(*n* = 1996)**	**Tertile 1** **(70.9–100, *n* = 662)**	**Tertile 2** **(100.1–104.7, *n* = 661)**	**Tertile 3** **(104.8–125.1, *n* = 673)**	***p*-value**
Chloride (mmol/L)	101.8 ± 6.0	95.2 ± 4.8^a,b^	102.5 ± 1.3^b^	107.6 ± 2.4	<0.001
Age ≥60 years	1,818 (91.1)	580 (87.6)^a,b^	616 (93.2)	622 (92.4)	<0.001
Male	840 (42.1)	246 (37.2)^a,b^	298 (45.1)	296 (44)	0.007
BMI (kg/m^2^)	20.8 (18.5, 23.4)	20.0 (18.1, 22.5)^a,b^	20.8 (18.4, 23.5)^b^	21.3 (19.0, 24.1)	<0.001
SBP (mmHg)	131.3 ± 24.2	124.1 ± 22.9^a,b^	132.1 ± 24.0^b^	137.4 ± 23.8	<0.001
DBP (mmHg)	76.7 ± 14.2	73.6 ± 13.6^a,b^	76.8 ± 13.9^b^	79.6 ± 14.4	<0.001
Heart rate (bpm)	82.0 (70.0, 98.0)	82.0 (70.0, 98.0)	81.0 (69.0, 99.0)	83.0 (71.0, 98.0)	0.797
NYHA class III or IV	1,649 (82.6)	560 (84.6)	536 (81.1)	553 (82.2)	0.227
Coronary artery disease	141 (7.1)	36 (5.4)	52 (7.9)	53 (7.9)	0.136
Diabetes	463 (23.2)	169 (25.5)^b^	159 (24.1)	135 (20.1)	0.049
Chronic kidney disease	472 (23.7)	180 (27.2)^a^	138 (20.9)	154 (22.9)	0.022
CCI ≥3	478 (23.9)	181 (27.3)^a,b^	147 (22.2)	150 (22.3)	0.046
LVEF (%) (*n* = 629)	50.7 ± 13.1	50.5 ± 13.2	50.7 ± 13.9	50.8 ± 12.3	0.964
LVEF ≥50%	347 (55.2)	97 (53.9)	123 (55.2)	127 (56.2)	0.898
LVEDD (mm) (*n* = 1,303)	53.2 ± 10.8	52.5 ± 11.8	54.0 ± 10.7	53.0 ± 9.9	0.135
E/A ratio (*n* = 522)	1.0 (0.7, 1.6)	0.9 (0.7, 1.7)	1.1 (0.7, 1.6)	0.9 (0.7, 1.6)	0.729
TRV(m/s) (*n* = 788)	3.0 ± 0.6	3.1 ± 0.7^b^	3.0 ± 0.6	2.9 ± 0.6	0.035
SCr (umol/L)	87.0 (64.9, 122.7)	89.8 (64.8, 127.2)	87.6 (65.4, 119.4)	85.5 (64.2, 115.8)	0.232
BUN (mmol/L)	8.0 (5.9, 11.5)	9.0 (6.3, 12.8)^a,b^	7.6 (5.8, 11.1)	7.5 (5.7, 10.6)	<0.001
eGFR (mL/min/1.73 m^2^)	64.8 (41.6, 90.1)	59.8 (39.2, 90.0)	64.6 (42.0, 89.4)	69.3 (44.8, 91.7)	0.098
Cystatin (mg/L)	1.6 (1.2, 2.2)	1.7 (1.3, 2.3)^a,b^	1.5 (1.2, 2.1)	1.5 (1.2, 2.1)	<0.001
Hematocrit (%)	0.4 ± 0.1	0.4 ± 0.1^a,b^	0.4 ± 0.1^b^	0.3 ± 0.1	<0.001
Hemoglobin (g/L)	115.1 ± 24.5	119.1 ± 24.9^a,b^	114.9 ± 23.9^b^	111.3 ± 24.2	<0.001
CO_2_CP (mmol/L)	23.8 ± 4.8	25.5 ± 5.5^a,b^	23.8 ± 4.2^b^	22.1 ± 4.1	<0.001
Phosphate (mmol/L)	1.1 ± 0.4	1.2 ± 0.6^a,b^	1.1 ± 0.4	1.1 ± 0.3	0.008
Potassium (mmol/L)	3.9 (3.5, 4.3)	3.9 (3.5, 4.4)	3.9 (3.6, 4.4)	3.9 (3.5, 4.3)	0.566
Hypokalemia	464 (23.2)	184 (27.8)^a,b^	144 (21.8)	136 (20.2)	0.003
Sodium (mmol/L)	139.0 (136.0, 141.4)	135.2 (132.0, 137.8)^a,b^	139.1 (137.0, 141.0)^b^	141.5 (139.6, 143.1)	<0.001
Hyponatremia	634 (31.8)	432 (65.3)^a,b^	156 (23.6)^b^	46 (6.8)	<0.001
BNP (pg/mL)	754.8 (308.4, 1739.0)	608.1 (208.3, 1660.0)^a,b^	770.2 (337.2, 1833.0)^b^	838.3 (397.5, 1740.0)	<0.001
Log BNP	9.6 (8.3, 10.8)	9.2 (7.7, 10.7)^a,b^	9.6 (8.4, 10.8)^b^	9.7 (8.6, 10.8)	<0.001
Number of event^*^	535 (26.8)	213 (32.2)^a,b^	165 (25.0)	157 (23.3)	<0.001

*BMI, body mass index; SBP, systolic blood pressure; DBP, diastolic blood pressure; HR, heart rate; NYHA, New York Heart Association; CCI, Charlson comorbidity index; LVEF, left ventricular ejection fraction; LVEDD, left ventricular end-diastolic diameter; TRV, tricuspid regurgitation velocity; SCr, serum creatinine; BUN, blood urea nitrogen; eGFR, estimated glomerular filtration rate; CO_2_CP, carbon dioxide combining power; BNP, brain natriuretic peptide; Log BNP: log-base-2 BNP*.

*: *number of patients meeting the composite outcome*.

a: *p < 0.05 compared with tertile 2*;

b: *p < 0.05 compared with tertile 3; p-value from Turkey or Games-Howell test*.

Patients in tertile 1 [including those with hypochloremia] were more likely to have diabetes or chronic kidney disease, higher NYHA class, higher Charlson comorbidity index, a higher rate of hypokalemia and hyponatremia compared to patients in tertile 2–3 (*p* < 0.01) (Table 1). Interestingly, the patients had lower BMI, lower SBP, lower history of coronary artery disease, lower eGFR and BNP.

### Chloride Levels and Clinical Outcomes

**Figure 2:**
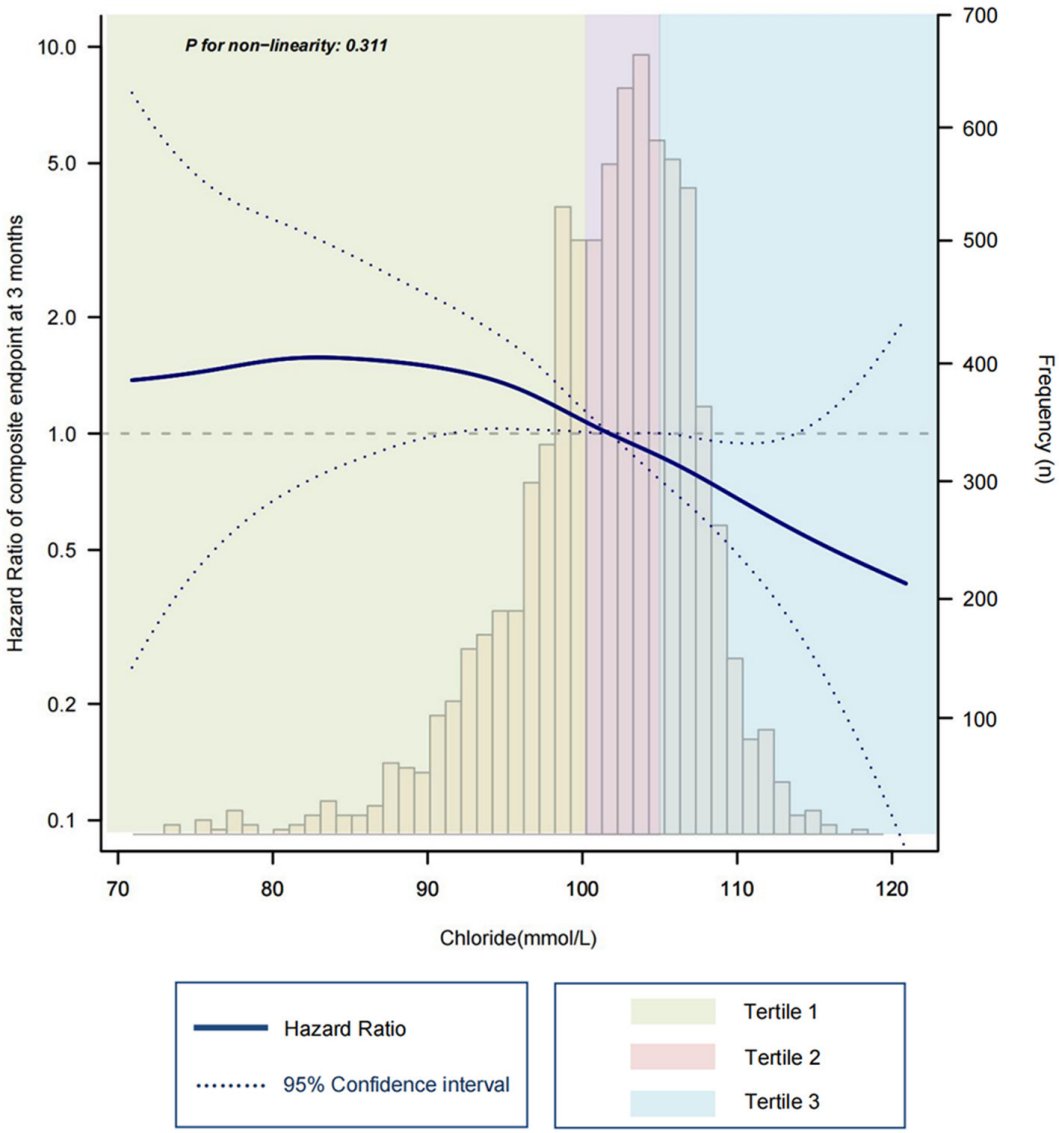
Spline plot for the associations of serum chloride with composite endpoint risk at 3 months. Hazard ratio (solid line) and 95% confidence intervals (dashed line) are estimated in a Cox proportional hazards model with adjustment for age, sex, NYHA class, diabetes, log BNP. Frequency bars show the proportion of patients with a specific chloride concentration.

The composite outcome occurred in 535 (26.8%) participants at 3 months. Over the same period, there were 213, 165, and 157 composite endpoint events for tertile 1 to 3, respectively. There was a highest event incidence rate of 32.2% in tertile 1 compared with that in tertile 2 and 3 (25.0 and 23.3%, respectively). The spline analysis supported a linear association with the composite endpoint over the range of chloride within this population (*p* = 0.311 for non-linearity, Figure 2). Similar linear association with serum chloride and readmission rate was also observed (*p* = 0.128 for non-linearity, Supplementary Figure S1). However, there seems to be a U-shaped relationship between serum chloride and all-cause mortality (*p* = 0.023 for non-linearity, Supplementary Figure S2).

The univariable and multivariable proportional hazards models (Table 2) showed that, admission chloride levels were inversely associated with the risk of composite endpoint. Each additional 1 mmol/L of chloride was associated with a 3% lower composite endpoint risk (HR, 0.970; 95% CI, 0.958 to 0.983; *p* < 0.001). After multivariate adjustment, this correlation remained stable (HR, 0.967; 95% CI, 0.939 to 0.996; *p* = 0.026).

**Table 2:** Univariable and multivariable cox proportional hazards models for composite endpoint.

	**Univariable**			**Multivariable**		
	**HR**	**95%CI**	***p*-value**	**HR**	**95%CI**	***p*-value**
Chloride (mmol/L)	0.970	0.958–0.983	<0.001	0.967	0.939–996	0.026
Age (yrs)	1.155	0.846–1.577	0.365			
Sex	0.897	0.756–1.064	0.212			
BMI	0.982	0.960–1.004	0.114			
SBP (mmHg)	0.990	0.986–0.993	<0.001			
DBP (mmHg)	0.990	0.984–0.996	0.002			
NYHA III/IV	1.784	1.364–2.334	<0.001	1.523	1.151–2.017	0.003
Coronary artery disease	0.992	0.710–1.385	0.962			
Diabetes	1.390	1.152–1.677	<0.001	1.274	1.045–1.552	0.016
Chronic kidney disease	1.554	1.293–1.867	<0.001			
LVEF (%)	0.996	0.985–1.008	0.554			
E/A ratio	1.226	0.960–1.565	0.102			
TRV (m/s)	0.973	0.775–1.222	0.815			
SCr (umol/L)	1.002	1.002–1.003	<0.001			
BUN (mmol/L)	1.050	1.037–1.063	<0.001			
eGFR (ml/min/1.73 m^2^)	0.993	0.991–0.996	<0.001			
Cystatin (mg/L)	1.238	1.151–1.331	<0.001			
Hematocrit (%)	0.169	0.051–0.567	0.004			
Hemoglobin (g/L)	0.995	0.992–0.999	0.007			
CO_2_CP (mmol/L)	0.967	0.956–0.984	<0.001			
Calcium (mmol/L)	1.159	0.725–1.853	0.537			
Potassium (mmol/L)	1.155	1.025–1.301	0.018			
Sodium (mmol/L)	0.953	0.939–0.968	<0.001			
Log BNP	1.111	1.056–1.168	<0.001	1.076	1.019–1.137	0.008

**Figure 3:**
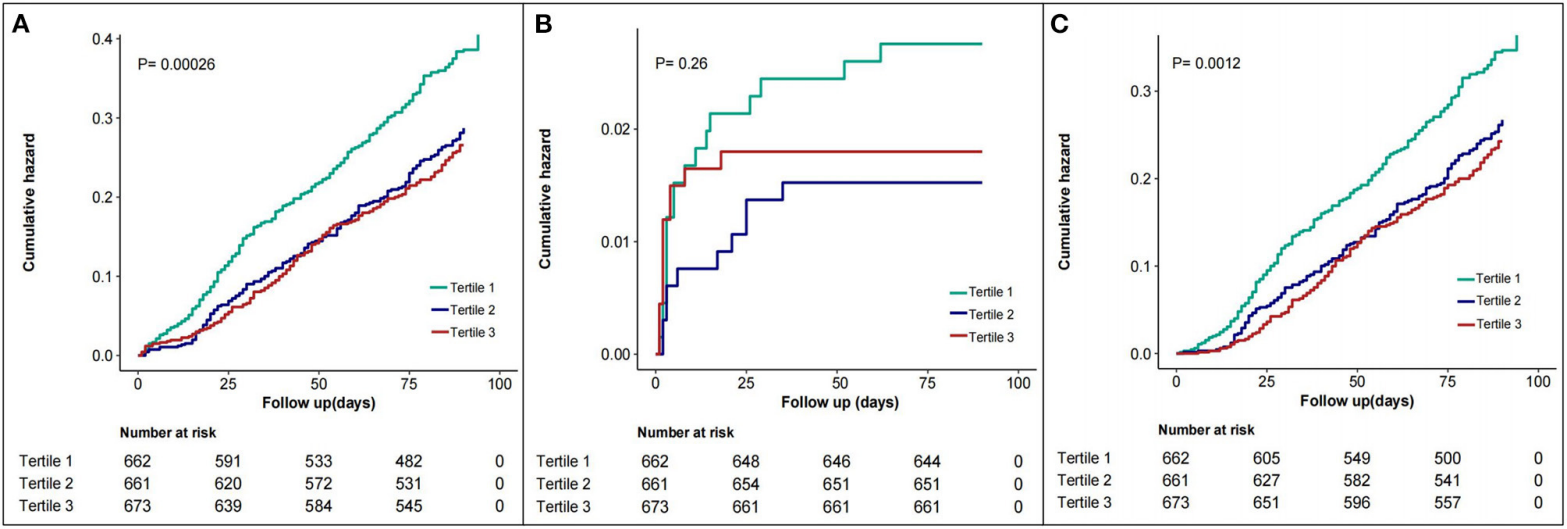
Kaplan–Meier estimates of 3 months outcomes according to serum chloride level. **(A)** for the composite endpoint event, **(B)** for all-cause death, **(C)** for all-cause rehospitalization. Chloride tertile1 is 70.9–100 mmol/L, tertile 2 is 100.1–104.7 mmol/L, and tertile 3 is 104.8–125.1 mmol/L.

Kaplan–Meier estimates of the cumulative incidence of the composite endpoint, all-cause death, and rehospitalization are shown in Figure 3 (log-rank *p* < 0.01, *p* = 0.26 and *p* < 0.01, respectively for Figures 3A–C).

### Serum Chloride and Sodium Levels

**Figure 4:**
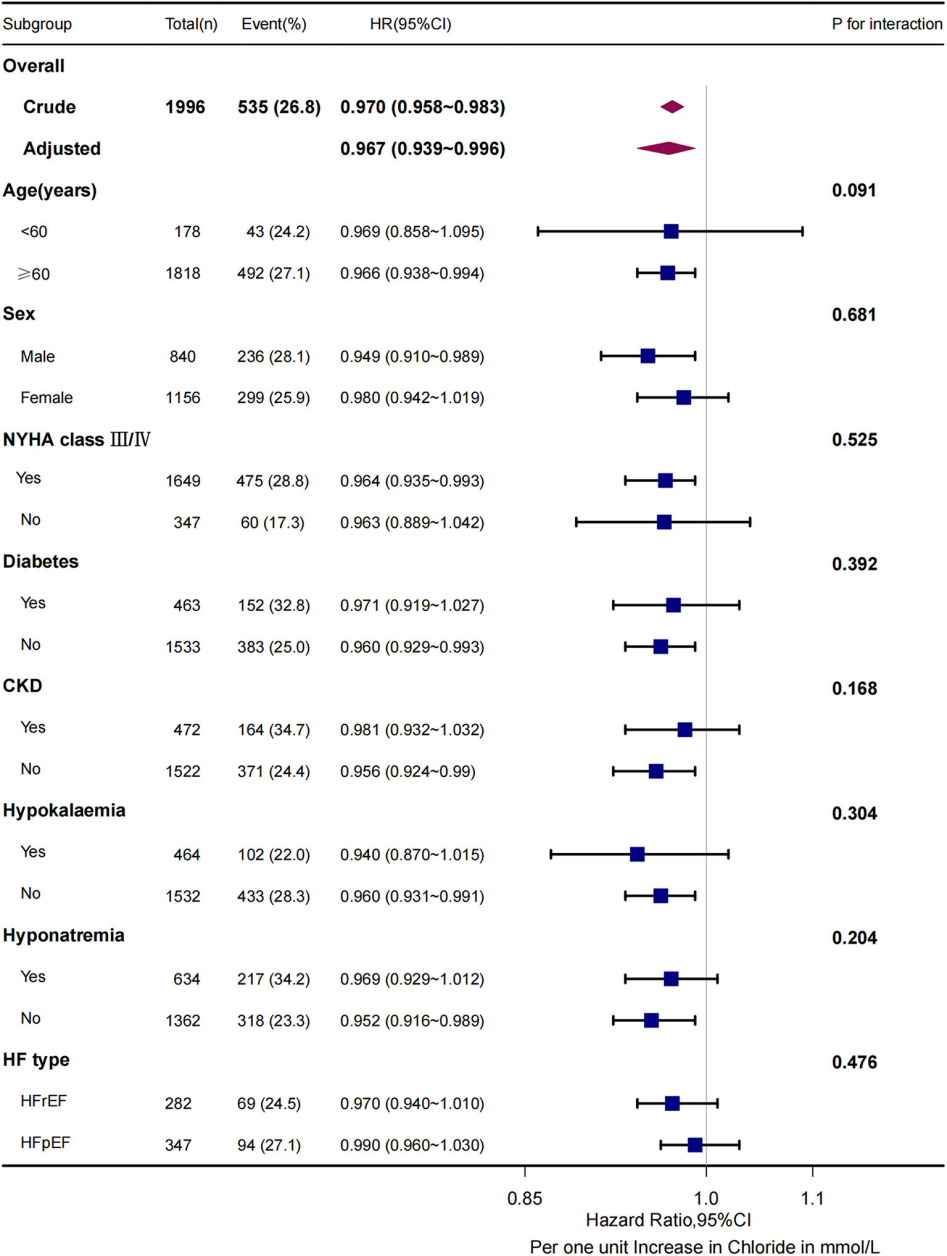
3-months composite endpoint risk across subgroups. For all subgroups, the P for interaction >0.05. CKD indicates chronic kidney disease; HFrEF, heart failure with reduced ejection fraction (LVEF <50%); HFpEF, heart failure with preserved ejection fraction (LVEF ≥50%).

Although sodium was also inversely associated with the risk of composite endpoint (HR, 0.953; 95% CI, 0.939 to 0.968; *p* < 0.001), after multivariable adjustment for other variables including chloride, sodium was not independently associated with the composite endpoint risk (HR, 1.007; 95% CI, 0.968 to 1.048; *p* = 0.713). Interestingly, Pearson correlation between serum chloride and sodium was 0.747 (*p* < 0.001) meaning that these variables potentially incorporate similar information. However, the ROC curve showed that AUC of model composed of age, sex, NYHA, diabetes, log BNP and chloride was 0.612; After adding sodium, AUC increased slightly and not significantly to 0.620 (*p* = 0.132, Supplementary Figure S3). Subgroup analyses found a consistent association between the chloride levels and risk of the composite endpoint across dichotomized subgroups of age (< or ≥60 years), sex, NYHA class (greater than class III), with or without diabetes, chronic kidney disease, hypokalemia, hyponatremia and heart failure type (heart failure with reduced ejection fraction (HFrEF) or preserved ejection fraction (HFpEF), Figure 4).

## Discussion

We selected a Chinese inpatient cohort with heart failure published in the PhysioNet database in 2021 to examine the relationship between serum chloride and prognosis. With the decrease of serum chloride level, BMI, BP, eGFR, BNP decreased, and the percentage of NYHA class III or above, the number of co-morbidities, hemoglobin, hematocrit, BUN, SCr, CO_2_CP, hypokalemia, and hyponatremia increased gradually. Importantly, lower serum chloride was associated with an increased risk of all-cause mortality or rehospitalization. This correlation remained stable after multivariable adjustment including serum sodium. In addition, subgroup analysis showed that the presence or absence of hyponatremia did not affect the negative association of serum chloride with the risk of the composite endpoint.

In this study, serum sodium <137 mmol/L was defined as hyponatremia. It was different to the traditional value, found in most studies, of 135 mmol/L. And it might interfere with the results of the multivariate regression. So, we used serum sodium concentration rather than hyponatremia in the multivariate regression analysis to avoid this bias. The lower history rate of coronary artery disease in the lowest chloride tertile was consistent with Zhang's study (15). However, the higher rate of Charlson comorbidity index above three points in this group might be risk factor of the adverse outcome.

Consistent with our results, previous studies demonstrated that admission serum chloride levels were independently and negatively associated with the risk of all-cause mortality or rehospitalization after multifactorial adjustment (10, 20–23). However, the data were either dated or with small sample sizes, mostly from Europe and USA, and the findings were not certain to be applicable to China. There were few studies on hypochloremia and heart failure in China. Our study extends these findings to Chinese hospitalized heart failure patients.

We found that low serum chloride was associated with adverse outcomes independently of serum sodium by multivariate Cox regression and ROC curve. However, correlation analysis of serum chloride and sodium showed a close correlation between them. This means the prognostic role of chloride might be affected by sodium. Previous studies have discussed the association and difference in prognostic ability between chloride and sodium concentrations in heart failure. However, there is still no clear conclusion. Some studies discovered chloride was independent of sodium for predicting high mortality in heart failure (10, 22). Others supported consideration of chloride and sodium interplay (15, 24). This difference may be related to study population, sample size, control of confounding factors, etc. Further studies are needed to better clarify this important interaction.

Although the underlying mechanisms of low serum chloride in the development of heart failure are not fully understood, some explanations can be hypothesized. Since chlorides are anions reabsorbed successively with sodium in the renal tubules, the possible mechanism of hypochloremia may be similar to the putative etiology of hyponatremia in heart failure patients (25). There are two mainstream statements: dilutional hypochloremia and depletive hypochloremia. The former is partly due to the reduced renal perfusion from the decreased cardiac output which stimulates the renin-angiotensin-aldosterone system as well as arginine vasopressin and sympathetic nervous system leading to fluid retention (14, 20). The latter is caused by the administration of diuretics, which inhibit the reabsorption of electrolytes in the thick ascending limb of the Henle loop and the distal convoluted tubule, promoting the excretion of chloride and leading to depletive hypochloremia. However, hypochloremia in turn induces renin secretion, increases the expression and activity of sodium-potassium-chloride cotransporters as well as sodium-chloride cotransporters, and promotes electrolytes reabsorption leading to diuretic resistance (25). Several studies have demonstrated that low serum chloride is apparently associated with neurohormonal activation and diuretic resistance, leading to impaired decongestive diuretic efficacy in patients with heart failure, which in turn increases the risk of death and readmission (26, 27). This may be a common consequence of hypochloremia.

Our results showed that in this Chinese inpatient heart failure cohort, low serum chloride at admission was associated with low BMI, low BNP, severe symptoms, high hematocrit, worsening renal function, and high co-morbidity. Ter Maaten JM et al. also found low baseline chloride was associated with high bicarbonate, poor diuretic response, and worsening heart failure (all *p* < 0.01) (20). These features are manifest after continuous administration of diuretics, and therefore the cause of hypochloremia is presumed to be depletion due to prehospital decongestive therapy. These findings are explainable. With a widespread implementation of heart failure guidelines in primary care clinics in recent years, the management of chronic heart failure has been dramatically improved. Out-of-hospital decongestion with oral diuretics has become routine. Hospitalization only occurs when oral diuretics fail. Therefore, most hospitalized heart failure patients, especially those with history of congestive heart failure, are in the second phase of decongestive therapy - removal of residual tissue congestion. The indicators of congestion at this stage are mostly high CA125, peripheral pitting edema, pulmonary rales, rather than high BNP, low hemoglobin, hematocrit and other indicators of intravascular congestion (28). Therefore, the prognostic role of serum chloride is not related to traditional prognostic indicators such as BNP or LVEF (regardless of heart failure type, HFrEF or HFpEF) (12). Meanwhile, we should recognize although it is common to attribute hypochloremia to diuretics use, some hypochloremia is not related to diuretics use. This has been reported in canine congestive heart failure patients where 24% of 1st time congestive heart failure admissions were hypochloremia in the absence of diuretic treatment (29). Therefore, recording hypochloremia in heart failure patients without diuretics will possibly reveal other mechanisms. Unfortunately, prehospital diuretics were not included in this dataset.

In addition, we found that the curve fit suggested a negative linear association between serum chloride and the risk of composite outcome; the similar relationship was observed between serum chloride and the risk of rehospitalization (Supplementary Figure S1). Interestingly, a U-shaped relationship was observed between serum chloride and all-cause mortality (Supplementary Figure S2). Our data suggests a decreased hazard ratio in the higher tertile, although this result must be taken cautiously, since only a small number of patients (*n* = 82) had hyperchloremia (chloride >110 mmol/L). However, this result may be valid, since it was supported also by previous studies (11). The pathophysiological explanation of this behavior is still difficult to provide and further studies are needed to clarify this issue.

The significant increase in mortality and readmission in patients with low serum chloride raises the question whether serum chloride levels may be a potential target for oral supplements or the use of chloramphetamine diuretics such as acetazolamide. Therefore, larger randomized studies are needed in the future to verify these findings and evaluate whether therapeutic maintenance of chloride homeostasis can improve the survival of patients with heart failure, or whether hypochloremia is simply a bystander marker of other processes.

There are several limitations in this study. First, because this was a single-center retrospective investigation, causality could not be determined. The patients in this study were all from Southwest China. Further research is needed to see if our findings remain true in different populations. Second, the follow-up period was short. Third, although some covariates are adjusted in the regression model, there may still be some unknown or unobtainable confounders. For example, medication at admission and chloride levels at discharge were not documented. Fourth, the interaction between chloride and sodium remains unclear in heart failure, which requires further analysis. Finally, the serum sodium concentration was determined by the indirect potentiometry, whose accuracy is inferior to that of direct potentiometry (30).

## Conclusions

In a southern Chinese hospitalized heart failure cohort, we found that admission serum chloride levels were independently and negatively associated with midterm all-cause mortality and readmission rate, and there was no interaction with serum sodium. Future research should focus on the pathophysiological mechanisms that cause hypochloremia and the prognostic impact of correcting hypochloremia.

## Data Availability Statement

Publicly available datasets were analyzed in this study. This data can be found here: https://doi.org/10.13026/8a9e-w734.

## Ethics Statement

The studies involving human participants were reviewed and approved by the Ethics Committee of Zigong Fourth People's Hospital. The Ethics Committee waived the requirement of written informed consent for participation.

## Author Contributions

Each author contributed substantially to the paper. ZF and XL conceived the study hypothesis. LA performed data analysis and ZF drafted the manuscript. LS and HL revised it critically for important intellectual content and supervised the writing of the manuscript. All authors approved the final version of the manuscript.

## Funding

This research was supported by the Key Science and Technology Foundation of China (2020YFC2004805) to LS.

## Conflict of Interest

The authors declare that the research was conducted in the absence of any commercial or financial relationships that could be construed as a potential conflict of interest.

## Publisher's Note

All claims expressed in this article are solely those of the authors and do not necessarily represent those of their affiliated organizations, or those of the publisher, the editors and the reviewers. Any product that may be evaluated in this article, or claim that may be made by its manufacturer, is not guaranteed or endorsed by the publisher.

## References

[1] Desai AS Stevenson LW. Rehospitalization for heart failure: predict or prevent? Circulation. (2012) 126:501–6. 10.1161/CIRCULATIONAHA.112.125435 22825412

[2] Roger VL. Epidemiology of heart failure: a contemporary perspective. Circ Res. (2021) 128:1421–34. 10.1161/CIRCRESAHA.121.318172 33983838

[3] Shafie AA Tan YP Ng CH. Systematic review of economic burden of heart failure. Heart Fail Rev. (2018) 23:131–45. 10.1007/s10741-017-9661-0 29124528

[4] Ambrosy AP Fonarow GC Butler J Chioncel O Greene SJ Vaduganathan M. The global health and economic burden of hospitalizations for heart failure. J Am Coll Cardiol. (2014) 63:1123–33. 10.1016/j.jacc.2013.11.053 24491689

[5] Girerd N Seronde M-F Coiro S Chouihed T Bilbault P Braun F. Integrative assessment of congestion in heart failure throughout the patient journey. JACC Heart Fail. (2018) 6:273–85. 10.1016/j.jchf.2017.09.023 29226815

[6] Rusinaru D Tribouilloy C Berry C Richards AM Whalley GA Earle N. Relationship of serum sodium concentration to mortality in a wide spectrum of heart failure patients with preserved and with reduced ejection fraction: an individual patient data meta-analysis: meta-Analysis Global Group in Chronic heart failure (MAGGIC). Eur J Heart Fail. (2012) 14:1139–46. 10.1093/eurjhf/hfs099 22782968

[7] Hauptman PJ Burnett J Gheorghiade M Grinfeld L Konstam MA Kostic D. Clinical course of patients with hyponatremia and decompensated systolic heart failure and the effect of vasopressin receptor antagonism with tolvaptan. J Card Fail. (2013) 19:390–7. 10.1016/j.cardfail.2013.04.001 23743487

[8] Felker GM Mentz RJ Cole RT Adams KF Egnaczyk GF Fiuzat M. Efficacy and safety of tolvaptan in patients hospitalized with acute heart failure. J Am Coll Cardiol. (2017) 69:1399–406. 10.1016/j.jacc.2016.09.004 27654854

[9] Nakao Y Saito M Yamaguchi O. Effect of tolvaptan on long-term prognosis in Japanese patients with heart failure: a systematic review and meta-analysis. Eur Heart J. (2020) 41(Supplement_2):1027. 10.1093/ehjci/ehaa946.1027

[10] Grodin JL Simon J Hachamovitch R Wu Y Jackson G Halkar M. Prognostic role of serum chloride levels in acute decompensated heart failure. J Am Coll Cardiol. (2015) 66:659–66. 10.1016/j.jacc.2015.06.007 26248993

[11] Cuthbert JJ Pellicori P Rigby A Pan D Kazmi S Shah P. Low serum chloride in patients with chronic heart failure: clinical associations and prognostic significance: chloride in chronic heart failure. Eur J Heart Fail. (2018) 20:1426–35. 10.1002/ejhf.1247 29943886

[12] Grodin JL Verbrugge FH Ellis SG Mullens W Testani JM Tang WHW. Importance of abnormal chloride homeostasis in stable chronic heart failure. Circ Heart Fail. (2016) 9:2453. 10.1161/CIRCHEARTFAILURE.115.002453 26721916 PMC4702267

[13] Yamaguchi S Abe M Iseki K Arakaki T Arasaki O Shimabukuro M. Prognostic impact of early changes in serum chloride concentrations among hospitalized acute heart failure patients - a retrospective cohort study. Circ Rep. (2020) 2:409–19. 10.1253/circrep.CR-20-0058 33693262 PMC7819648

[14] Kataoka H. Chloride in heart failure syndrome: its pathophysiologic role and therapeutic implication. Cardiol Ther. (2021) 10:407–28. 10.1007/s40119-021-00238-2 34398440 PMC8555043

[15] Zhang Y Peng R Li X Yu J Chen X Zhou Z. Serum chloride as a novel marker for adding prognostic information of mortality in chronic heart failure. Clin Chim Acta Int J Clin Chem. (2018) 483:112–8. 10.1016/j.cca.2018.04.028 29684381

[16] Zhang Z Cao L Chen R Zhao Y Lv L Xu Z. Electronic healthcare records and external outcome data for hospitalized patients with heart failure. Sci Data. (2021) 8:1–6. 10.1038/s41597-021-00835-9 33547290 PMC7865067

[17] Goldberger AL Amaral LA Glass L Hausdorff JM Ivanov PC Mark RG. PhysioBank, PhysioToolkit, and PhysioNet: components of a new research resource for complex physiologic signals. Circulation. (2000) 101:E215–220. 10.1161/01.CIR.101.23.e215 10851218

[18] Ponikowski P Voors AA Anker SD Bueno H Cleland JGF Coats AJS. 2016 ESC Guidelines for the diagnosis and treatment of acute and chronic heart failure: the task force for the diagnosis and treatment of acute and chronic heart failure of the European society of cardiology (ESC) developed with the special contribution of the heart failure association (HFA) of the ESC. Eur Heart J. (2016) 37:2129–200. 10.1093/eurheartj/ehw128 27206819

[19] Ouwerkerk W Voors AA Zwinderman AH. Factors influencing the predictive power of models for predicting mortality and/or heart failure hospitalization in patients with heart failure. JACC Heart Fail. (2014) 2:429–36. 10.1016/j.jchf.2014.04.006 25194294

[20] Ter Maaten JM Damman K Hanberg JS Givertz MM Metra M O'Connor CM. Hypochloremia, diuretic resistance, and outcome in patients with acute heart failure. Circ Heart Fail. (2016) 9:e003109. 10.1161/CIRCHEARTFAILURE.116.003109 27507112

[21] Grodin JL Sun JL Anstrom KJ Chen HH Starling RC Testani JM. Implications of serum chloride homeostasis in acute heart failure (from ROSE-AHF). Am J Cardiol. (2017) 119:78–83. 10.1016/j.amjcard.2016.09.014 27816115 PMC5161696

[22] Marchenko R Sigal A Wasser TE Reyer J Green J Mercogliano C. Hypochloraemia and 30 day readmission rate in patients with acute decompensated heart failure. ESC Heart Fail. (2020) 7:903–7. 10.1002/ehf2.12587 32286008 PMC7261563

[23] Misumi K Matsue Y Nogi K Sunayama T Dotare T Maeda D. Usefulness of incorporating hypochloremia into the get with the guidelines-heart failure risk model in patients with acute heart failure. Am J Cardiol. (2022) 162:122–8. 10.1016/j.amjcard.2021.09.020 34763832

[24] Ferreira JP Girerd N Duarte K Coiro S McMurray JJV Dargie HJ. Serum chloride and sodium interplay in patients with acute myocardial infarction and heart failure with reduced ejection fraction: an analysis from the high-risk myocardial infarction database initiative. Circ Heart Fail. (2017) 10:e003500. 10.1161/CIRCHEARTFAILURE.116.003500 28159825

[25] Berend K van Hulsteijn LH Gans ROB. Chloride: the queen of electrolytes? Eur J Intern Med. (2012) 23:203–11. 10.1016/j.ejim.2011.11.013 22385875

[26] Clinical significance of spot urinary chloride concentration measurements in patients with acute heart failure: investigation on the basis of the ‘tubuloglomerular feedback’ mechanism. Cardiol Open Access. (2021) 6:04. 10.33140/coa.06.01.04

[27] Plasma renin activity after diuretic treatment in patients with stable heart failure: with special reference to its association with electrolyte chloride. Cardiol Open Access. (2021) 6:01. 10.33140/coa.06.02.01

[28] Boorsma EM ter Maaten JM Damman K Dinh W Gustafsson F Goldsmith S. Congestion in heart failure: a contemporary look at physiology, diagnosis and treatment. Nat Rev Cardiol. (2020) 17:641–55. 10.1038/s41569-020-0379-7 32415147

[29] Roche-Catholy Marine Van Cappellen Iris Locquet Laurent. Clinical relevance of serum electrolytes in dogs and cats with acute heart failure: a retrospective study. J Vet Intern Med. (2021) 35:1652–62. 10.1111/jvim.16187 34096660 PMC8295692

[30] Milani GP Bianchetti MG Lava SA. Measurement of sodium in heart failure. Heart. (2018) 104:1724. 10.1136/heartjnl-2017-312799 30262637

