# Supplementary material for: Serum Chloride Is Inversely Associated With 3 Months Outcomes in Chinese Patients With Heart Failure, a Retrospective Cohort Study
Source: Front Cardiovasc Med. 2022 Apr 28;9:855053. doi: 10.3389/fcvm.2022.855053 (PMC9096445; doi:10.3389/fcvm.2022.855053)
Supplement: Supplementary file 1 [file Data_Sheet_1.docx]

**Supplementary Figures**


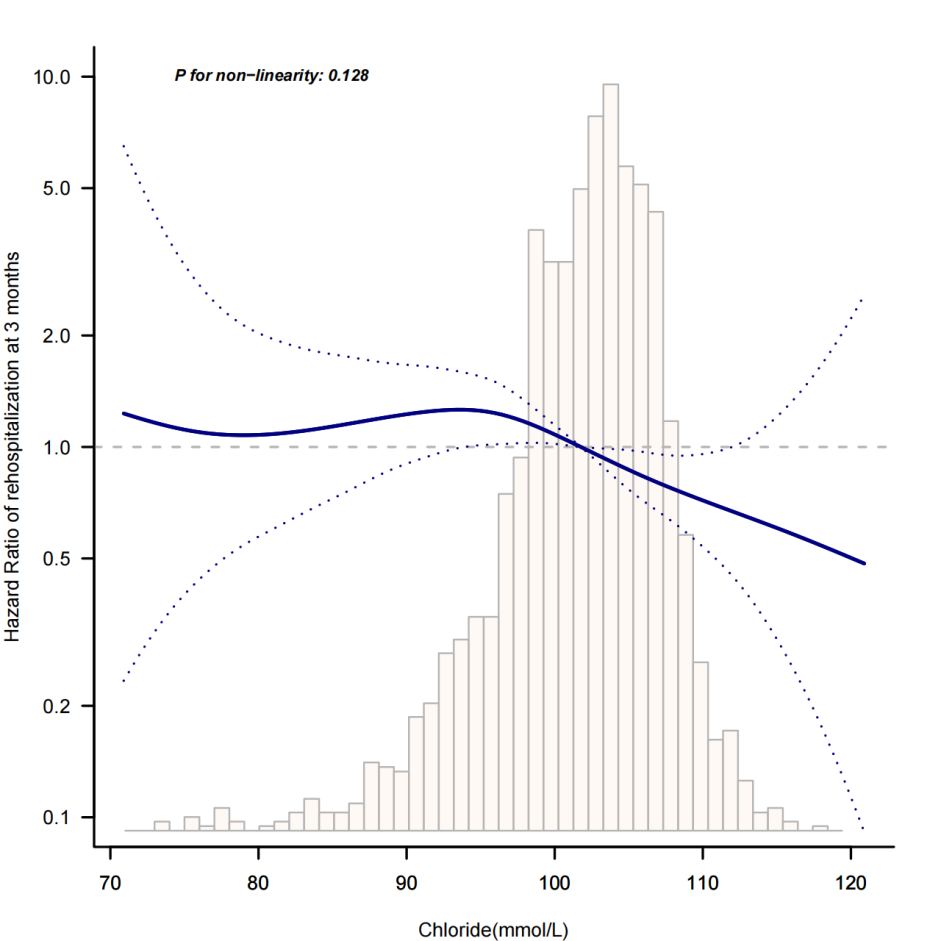


**Figure S1.** Spline plot for the associations of serum chloride with the risk of rehospitalization at 3 months. Hazard ratio (solid line) and 95% confidence intervals (dashed line) are estimated in a Cox proportional hazards model with no adjustment for covariants. Frequency bars show the proportion of patients with a specific chloride concentration.


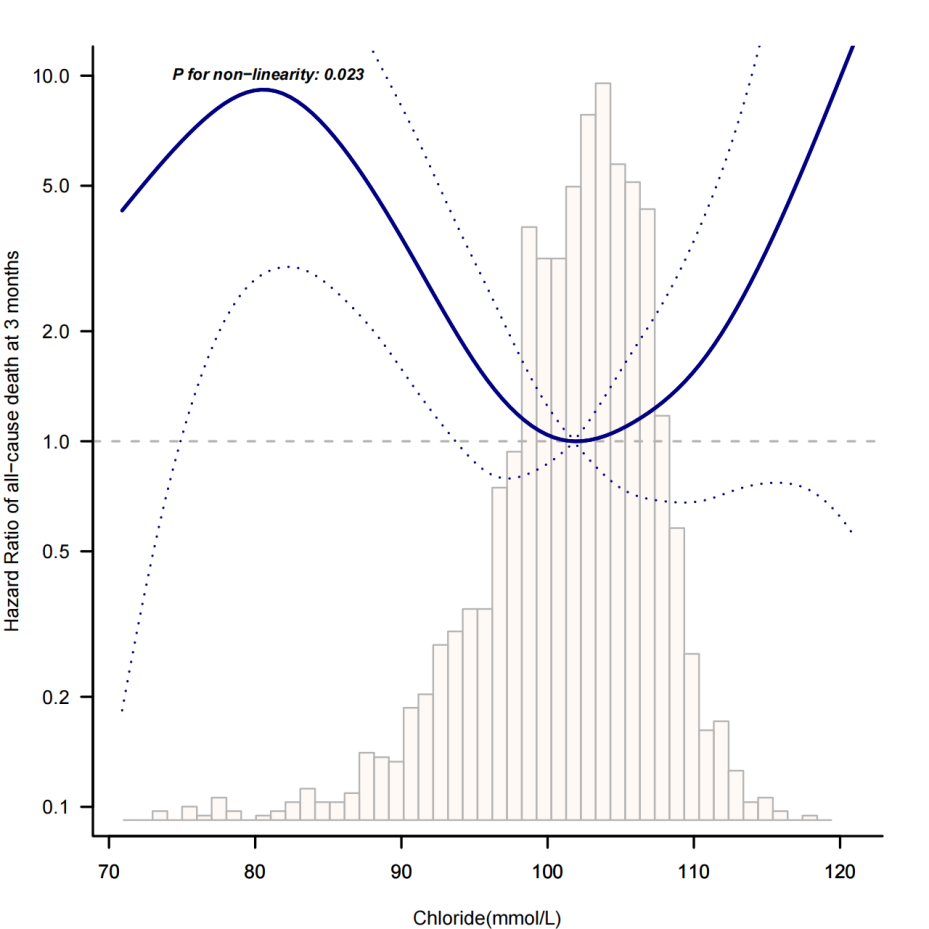


**Figure S2.** Spline plot for the associations of serum chloride with the risk of all-cause death at 3 months. Hazard ratio (solid line) and 95% confidence intervals (dashed line) are estimated in a Cox proportional hazards model with no adjustment for covariants. Frequency bars show the proportion of patients with a specific chloride concentration.


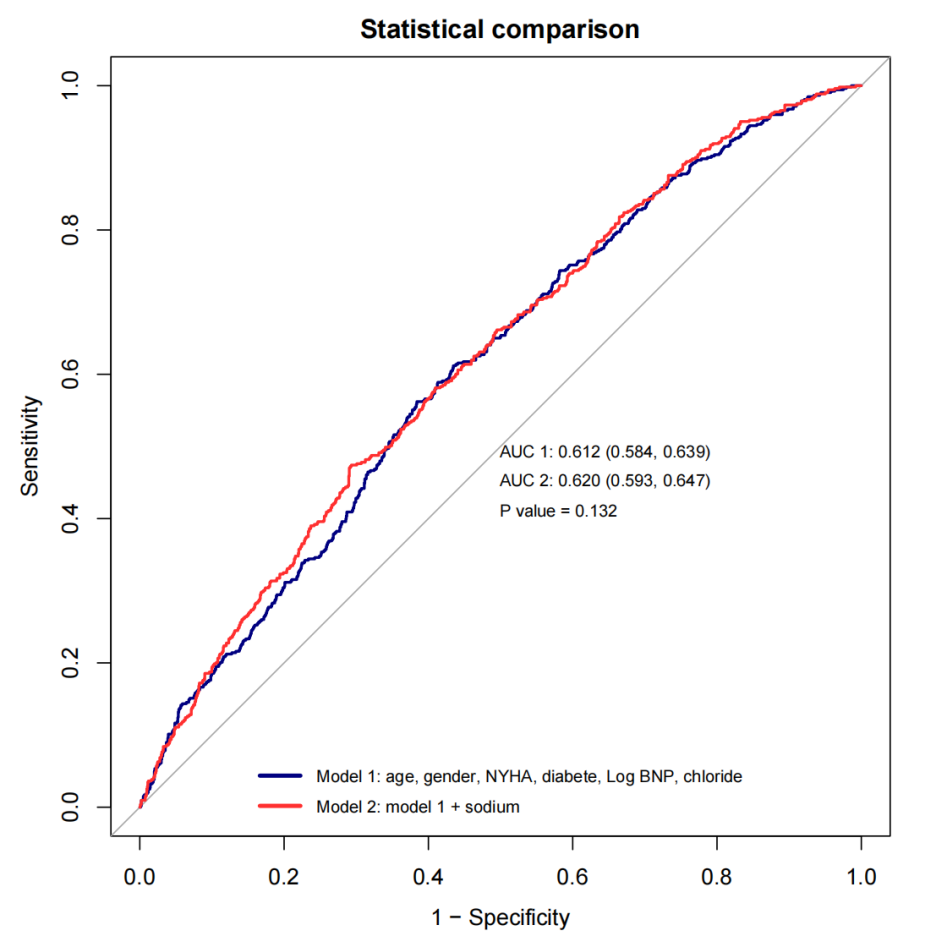


**Figure S3.** Receiver-operating curve of chloride levels for predicting composite endpoint of patients with HF. The AUC of model 1(composed of age, gender, NYHA, diabetes, log BNP and chloride) was 0.612; After adding sodium, AUC increased slightly to 0.620. There was no significant difference between two model (P=0.132). AUC = area under the curve.
